# The Costs, Benefits and Human Behaviours for Antimicrobial Use in Small Commercial Broiler Chicken Systems in Indonesia

**DOI:** 10.3390/antibiotics9040154

**Published:** 2020-04-01

**Authors:** Lucy Coyne, Ian Patrick, Riana Arief, Carolyn Benigno, Wantanee Kalpravidh, James McGrane, Luuk Schoonman, Ady Harja Sukarno, Jonathan Rushton

**Affiliations:** 1 Epidemiology and Population Health, University of Liverpool, Neston CH64 7TE, UK; ianpatrick4229@gmail.com (I.P.); jrushton@liverpool.ac.uk (J.R.); 2 Agricultural and Resource Economic Consulting Services, Armidale, NSW 2350, Australia; 3 Center for Indonesian Veterinary Analytical Studies, Bogor 16310, Indonesia; rianaarief83@gmail.com; 4 Regional Food and Agriculture Organization (FAO) Office for Asia and the Pacific, Bangkok 10200, Thailand; carolynbenigno@gmail.com (C.B.); Wantanee.Kalpravidh@fao.org (W.K.); 5 Food and Agriculture Organization (FAO) Country Office for Indonesia, Jakarta 10250, Indonesia; James.McGrane@fao.org (J.M.); luuk.schoonman@gmail.com (L.S.); adiharja@gmail.com (A.H.S.)

**Keywords:** chicken production, antimicrobial use, antimicrobial resistance (AMR), Indonesia, antibiotic, economics, behaviours

## Abstract

There are growing concerns over the threat to human health from the unregulated use of antimicrobials in livestock. Broiler production is of great economic and social importance in Indonesia. This study used a structured questionnaire approach to explore the human behaviours and economic drivers associated with antimicrobial use in small commercial broiler systems in Indonesia (*n* = 509). The study showed that antimicrobial use was high with farmers easily able to access antimicrobials through local animal medicine, however, it was difficult for farmers to access veterinary advice on responsible antimicrobial use. The most significant finding was that the relative cost of antimicrobials was low, and farmers observed improvements in productivity rates from routine antimicrobial administration. However, farmers seldom kept detailed records on farm productivity or economic costs; this is a hurdle to undertaking a more detailed economic analysis of antimicrobial use. There is a need for further research on the cost-effectiveness of alternative methods of preventing disease and ensuring that feasible alternatives are easily available. Farm-level economics and securing the food supply chain need to be central to any future policy interventions to reduce antimicrobial use in broiler systems in Indonesia and this observation is relevant at a regional and global level.

## 1. Introduction

Antimicrobial resistance (AMR) poses a risk to global health by threatening effective infectious disease treatments [1]. The World Health Organization (WHO) consider it to be one of the ten greatest threats to human health and have made it a priority area in their 13th General Programme of Work (2019–2023) [2,3]. This stark view is supported by estimates that if AMR is left unchecked, it will lead to an additional 10 million early deaths by 2050 at an economic cost of United States Dollar (USD) 100 trillion [4]. 

It has long been suspected that indiscriminate use of antimicrobials in human and animal health would lead to the development of AMR through selection pressures in bacterial populations for the development and exchange of resistance genes [5]. There is growing evidence that antimicrobial use in livestock plays an important role in driving resistance in humans through the zoonotic spread of resistant bacteria [6]. Resistance may spread to humans through either direct contact with animals, meat consumption, contamination of vegetable matter with manure or indirectly through environmental pathways [7,8,9,10]. At present, this risk is unquantified, however, research into intensive animal production systems has identified that the intestinal microbiota of livestock can act as a source of resistant bacteria for consumers, farmers and those people living in close proximity to animals [7,8,11,12]. 

On a global scale, antimicrobials are widely used in livestock production for both disease prevention and growth promotion [10]. The majority of antimicrobial classes are equally used in both human medicine and animal production with data from the United States estimating that around 70% of the antimicrobials used in animals are of medical importance to human medicine [13]. Thus, bacterial populations in both human and animal reservoirs face parallel selection pressures from shared antimicrobial classes. AMR does not respect species barriers or country borders and therefore requires a uniform and harmonised approach from the human, environmental and animal health perspectives. There is great need for antimicrobials to be viewed as a global public good [14] and for AMR to be tackled using a collaborative, One Health route [15,16]. 

The use of antimicrobial growth promoters (AGPs) has been prohibited in the European Union since 2006 [17]. Long term there have been minimal negative effects on health and productivity; however, in the short term some countries observed a slight increase in antimicrobial use for therapeutic reasons, believed to be in response to clinical disease previously masked by the use of AGPs [18]. Whilst this success is to be commended, it is an example from high-income industrialized countries with well-developed infrastructures for livestock production. An economic analysis showed that such bans would have negative economic consequences in low- and middle-income countries with less optimised production systems such as Indonesia [19].

Indonesia has an estimated human population of 263 million, which continues to grow, and presents one of the most rapidly expanding consumer markets in the world [20,21]. In 2013, agriculture accounted for around 12% of the gross domestic product and employed around 42 million people, accounting for around 40% of the total workforce [22]. Broiler chickens are the most important source of animal protein accounting for 87% of total meat consumption. The Indonesian Feed Producers Association (APPI/GPMT) has reported that the broiler sector provides jobs for 12 million people with the national broiler flock in the region of 3.5 billion, making it important both socially and economically [23]. Due to the large number of people involved in the production and processing chain alongside the numbers of consumers, there is a significant AMR risk from the misuse of antimicrobials. Thus, there is a need to weigh the risk against the benefits of this production and marketing system.

The threat AMR poses to human health in Indonesia remains largely unknown, however, rates of AMR are thought to be high and present a growing challenge to public health [24]. The results from an AMR surveillance study in twelve Asian-Pacific region countries identified that Indonesia had the highest rate of extended spectrum beta-lactamase (ESBL) positive *E. coli* (71%) and *Klebsiella* spp. (64%) amongst the nations investigated [25]. Initial information on antimicrobial use and AMR levels in livestock indicates that rates of both are high [10,24,26]. Van Boeckel et al. [10] highlighted Indonesia as one of five countries most likely to show the greatest increase in antimicrobial consumption globally with an estimated increase of 202% by 2030 from 2010 levels. This estimate reflects the use of the model based on changes in human population, demand for animal protein and the type of livestock system, rather than empirical data. 

Empirical studies estimated that antimicrobial consumption is high in Indonesian broiler production and accounts for around 60% of all use in livestock species (60%) [27,28,29,30]. This high use appears to be associated with high AMR levels in broiler flocks with Usui et al. [31] identifying high levels of resistance to tetracyclines, fluoroquinolones and penicillins from commensal *E.coli* in Indonesian broilers and Yulistiani et al. [32] finding that 61% of the Enterobacteriaceae strains isolated from poultry meat sourced from wet markets in Surabaya city were multi-drug resistant. Whilst the literature identifies a parallel trend for both high antimicrobial use and AMR, knowledge gaps remain on antimicrobial use behaviours and the economic importance of use in the Indonesian broiler sector. The current study explores the costs and benefits of antimicrobial use in the small commercial broiler sectors which are likely to be similar at a global level. The focus of this work was in the Lampung, Central Java and West Kalimantan provinces in Indonesia. 

## 2. Results

### 2.1. Respondent and Farm Information

The survey encompassed a total of 509 small-scale commercial broiler farms across three provinces in Indonesia: Lampung (*n* = 51), Central Java (*n* = 165) and West Kalimantan (*n* = 293). Background information on the study respondents and characteristics of the broiler farms are shown in Table 1 and Table 2. 

Just over half (57%) of the farmers were producers who had an established contract with a large integrated company. It has been reported that such an approach minimises the risk of high production costs in the face of a declining market and offers a more secure income for farmers [33]. Typically, in these contract agreements the contractor provides day old chicks (DOCs), feed, antimicrobials, vaccines, technical advice and product marketing while the farmers provide the housing and labour [34]. There were substantial differences in farm type between the provinces with 78% and 97% of farms in Lampung and Central Java province being contract farms respectively, in comparison to only 28% in West Kalimantan province. Contrasts were also observed in the flock size with West Kalimantan having a lower median flock size (2000) in comparison to either Lampung (8000) or Central Java (5000) provinces. Farms across all provinces were predominantly open house systems (97%). A traditional open house is a shed with a high roof, natural ventilation, manual feeding and watering systems and open-sided walls which may or may not be covered with netting [35].

### 2.2. Perceptions on AMR and Antimicrobial Use Practices

The WHO definition of AMR is ‘the ability of a microorganism (like bacteria, viruses, and some parasites) to stop an antimicrobial (such as antibiotics, antivirals and antimalarials) from working against it. As a result, standard treatments become ineffective, infections persist and may spread to others’ [36]. Questionnaire respondents were asked to describe what they understood by the term AMR and only 42% (*n* = 509) were able to show a basic understanding of AMR or its impacts. The two most common definitions were that AMR resulted in ‘resistance to antibiotic/drugs’ (reported by 21%), although it was not specified whether the subject was the disease or the chicken, and ‘Treatment becomes ineffective’ (reported by 19%). 

**Figure 1:**
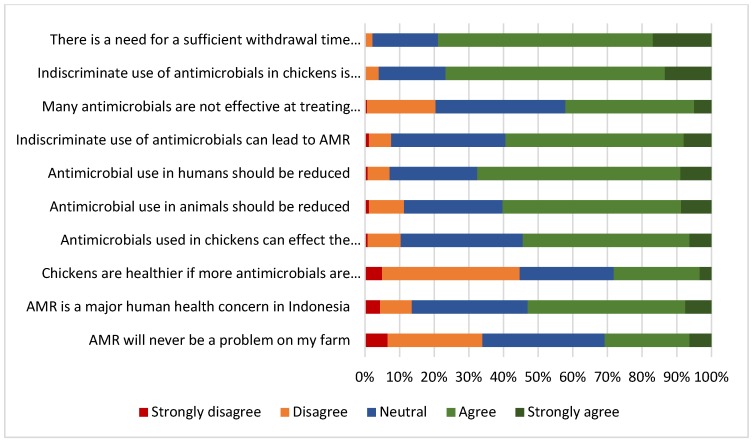
Respondent opinion on the importance of antimicrobial resistance (AMR) concerns and the role of antimicrobial use in broilers in Indonesia (*n* = 509)**.**

The questionnaire sought farmer opinion on the importance of AMR concerns and the role of antimicrobial use practices in broiler production (Figure 1). More than two thirds of respondents agreed that the indiscriminate use of antimicrobials could result in high costs for farmers (76%) and 79% identified that it was necessary to adhere to antimicrobial withdrawal times before harvesting broilers for slaughter. The majority of farmers also identified that AMR is a major human health concern in Indonesia (53%), that antimicrobial use in chickens can affect the health of the consumer (54%), that the indiscriminate use of antimicrobials can lead to AMR (58%) and that antimicrobial use should be reduced in human (67%) and veterinary medicine (61%). Conversely, the majority of respondents did not agree that the health of chickens improves if more antimicrobials are used (45%). More than a quarter (30%) of the farmers held the opinion that AMR will never be a problem on their farms. Overall opinion was divided on whether antimicrobials were effective in treating disease. This divided opinion fits with the issue that antimicrobials are often applied to diseases that are viral [37].

There were some significant differences observed between the attitudes of contract and independent farmers to AMR and antimicrobial use behaviours (univariate tables are shown in Table S1). Independent farmers more frequently held the opinion that the indiscriminate use of antimicrobials was expensive (*p* ≤ 0.0005), that withdrawal times were necessary (*p* = 0.001), that using more antimicrobials would not necessarily improve chicken health (*p* ≤ 0.0005) and that treatment failure was an issue in chickens (*p* = 0.014) compared with contract farmers. In addition, more independent farmers felt that antimicrobial use should be reduced in animals (*p* ≤ 0.0005) and humans (*p* ≤ 0.0005) when compared with contract farmers. In contrast, contract farmers were significantly less likely to be able to correctly define AMR when compared with independent farmers (*p* = 0.046).

### 2.3. Drivers for Antimicrobial Use

**Figure 2:**
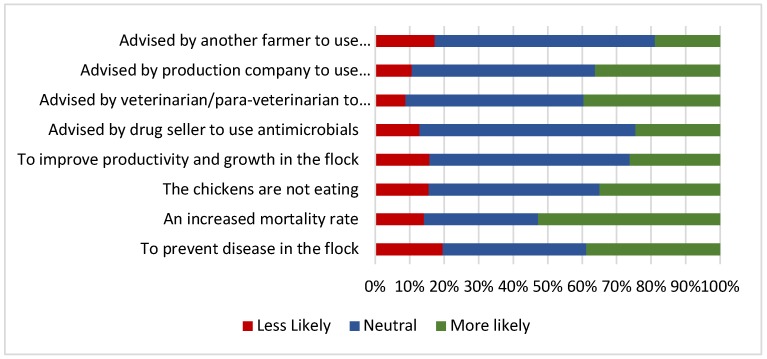
Drivers for antimicrobial use by broiler farmers in the small commercial broiler sector in Indonesia (*n* = 509).

The study also explored drivers for antimicrobial use by broiler farmers (Figure 2). The majority of respondents reported that an increased mortality rate (52%) would lead to increased antimicrobial use. In addition, 39% of respondents identified that disease prevention was a motivation for antimicrobial use. Conversely, a minority identified that inappetence in the flock (35%) and improving growth and productivity (26%) would influence their antimicrobial use behaviours. Advice from drug sellers, veterinarians/para-veterinarians, the contract company and other farmers was not considered to either positively or negatively influence antimicrobial use behaviours by the majority of respondents. There were some significant differences between the drivers of antimicrobial use in contract and independent farmers. Contract farmers were significantly more likely to report using antimicrobials to prevent disease (*p* = 0.036) and to use antimicrobials based on advice from a contract company (*p* ≤ 0.0005) when compared with independent farmers (*p* = 0.036).

**Figure 3:**
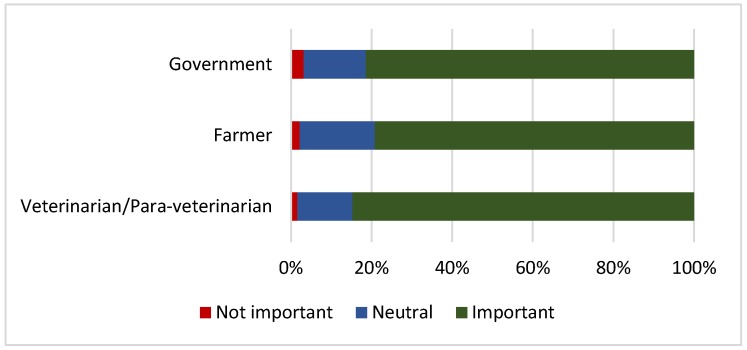
Respondents’ opinion on the roles of different actors in monitoring the responsible use of antimicrobials in broilers (*n* = 509)**.**

Respondents were asked to consider the role of different actors in monitoring the responsible use of antimicrobials in broilers (Figure 3). The majority of respondents considered that veterinarians/para-veterinarians (85%), the government (81%) and farmers themselves (79%) were important in monitoring the responsible use of antimicrobials in broilers. There were some significant differences observed between the contract and independent farmers’ opinions on the roles of different actors. Contract farmers were significantly more likely to consider veterinarians (*p* = 0.001) and the government (*p* ≤ 0.0005) to have a neutral role in monitoring the responsible use of antimicrobials in broilers in comparison to independent producers. 

### 2.4. Perceptions on the Economics of Antimicrobial Use

Farmers were asked to consider the economic importance of antimicrobials to broiler production on their farms. The majority of respondents (88%, *n* = 502) reported that a health issue in the broiler flock would have a negative impact on farm profit. The majority of farmers reported that they recorded some data relating to farm productivity (77%, *n* = 435). The data most commonly captured across the farms related to body weight (38%, *n* = 324) and mortality rates (38%, *n* = 324). Other productivity parameters recorded included sale price (16%, *n* = 324), feed intake levels (6%, *n* = 324) and feed costs (2%, *n* = 324).

The majority (82%, *n* = 501) of farmers believed that using antimicrobials provided them with an economic advantage. Contract farmers were significantly more likely to identify an economic advantage to antimicrobial use compared with independent farmers (*p* ≤ 0.0005). Farmers considered increased productivity (29%, *n* = 490) and maintaining the health of their flock (25%, *n* = 490) as the most important reasons for using antimicrobials. Farmers also believed that antimicrobial use reduced mortality rates (20%, *n* = 490) and could be used to prevent (18%, *n* = 490) and treat (8%, *n* = 490) disease. 

### 2.5. Costs of Production

The economic analysis is based on data collected from farmer recall of the last completed broiler production cycle. A production cycle is usually between 28 to 33 days and there can be up to seven cycles per year. In a cross-sectional survey such as this, responses were sometimes not provided by the farmers; this was either because they could not recall the answer or were unwilling to divulge their information. In the following cost of production analysis any minor gaps in farm data were filled in with district averages. The highest costs were in the West Kalimantan districts of Ketapang and Mempawah with USD 2.97 and USD 2.84 per bird, respectively. The lowest average costs were in Sekadau, also in West Kalimantan, at USD 1.79 per bird (see Table 3). 

As would be expected, the major cost items in broiler production were DOCs and feed; medicines, vaccines and disinfectants were very minor components of the cost structure. It has not been possible to separate antimicrobials from other medicines such as vitamins and probiotics, etc. Even so, the total cost of these medicines/drugs is low. They range from 0.7% of total costs in Mempawah to 2.9% in Ketapang. 

The size of the farm influenced the overall costs, but the results were not consistent in all areas. In West Kalimantan, the area with the greatest number of small farms surveyed, the overall costs were higher in small farms than in large farms. Although in Central Java there was no difference between the costs of large and small farms, and in Lampung the costs in small farms were less than for large farms (Table S2). 

The data also allowed some distinction between contract and independent farms. In general, the costs on the contract farms were lower than for independent farms, but again there were differences between the regions. In West Kalimantan it was the independent farms who had greater costs, whereas in Lampung it was the contract farms. A summary of these data can also be found in Table S3.

### 2.6. Relationships between Farm Size, Management Type, Productivity and Antimicrobial Use

Data were explored to look at two different aspects: (1) the relationship between farm size, farm management type (independent or contract) and antimicrobial use and (2) farm productivity and antimicrobial use. Antimicrobial use is represented as the number of days per batch that antimicrobials were administered, a more accurate estimate of actual antimicrobial use was not possible to attain through the survey instrument. 

**Figure 4:**
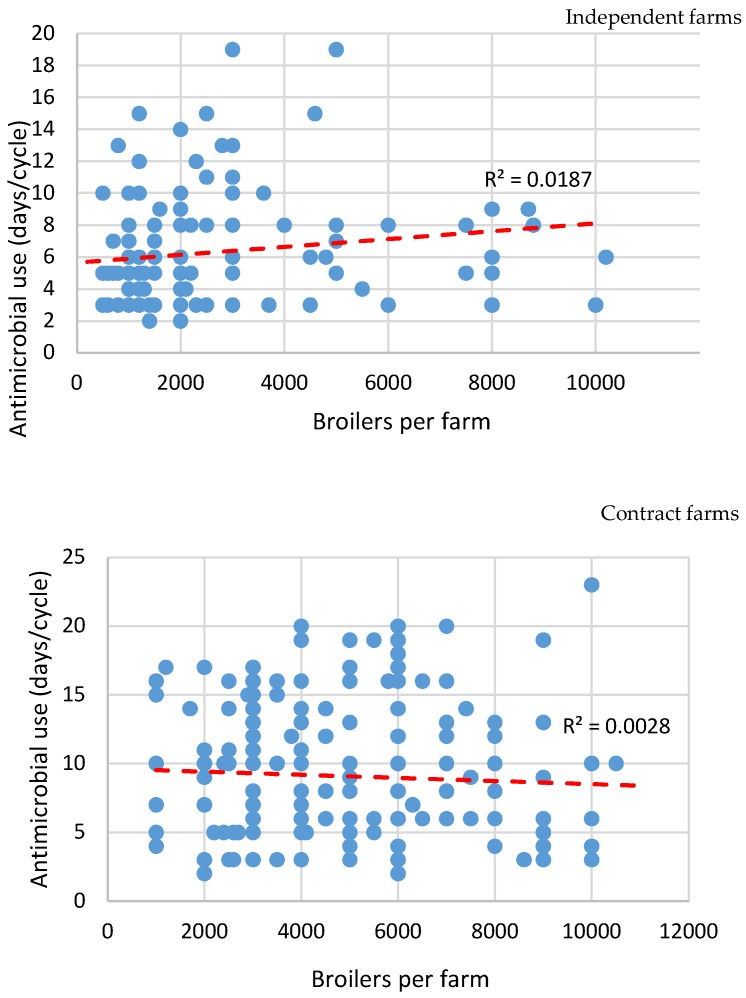
The correlation between number of days antibiotics given, farm size and management type (contract vs. independent).

There was a total of 363 farms that provided estimates of the number of days that they provided antimicrobials to their latest batch of birds, 150 of these were independent farmers and the remaining 213 were contract farmers. Each data point in Figure 4 represents a farmer’s estimate of days that antimicrobials were used in the last batch of broilers. 

Initial analysis indicated a weak association between flock size and number of days reported where antimicrobials were used. In smaller flocks (>3000 birds) antimicrobials may be used more frequently than in larger flocks. When considered from the perspective of flock size and farm management type, there was an indication of increasing antimicrobial use on independent farms with larger flocks. Farm size made no apparent difference in the contract farms. The association was weak (see Figure 4).

**Figure 5:**
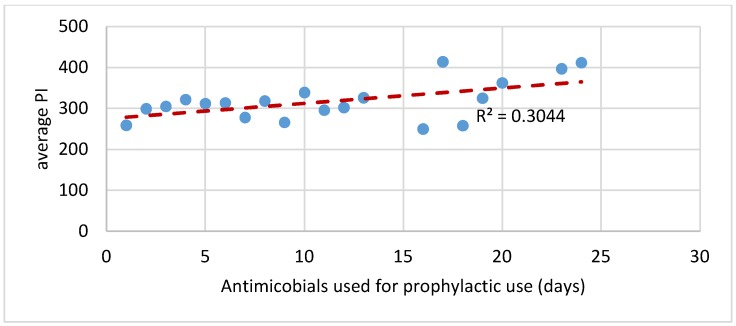
The correlation between average performance index (PI) and antimicrobials use for prophylactic reasons.

Further analysis was carried out to evaluate the relationship between production and antimicrobial use. Production was measured by calculating the performance index (PI) for each batch. The PI combines weight at sale, mortality rate and feed conversion ratio to produce a metric that measures batch performance. The higher the PI the better the performance of the batch or cycle of broilers. When calculated and plotted against the number of days that antimicrobials were reported to be used, there was a positive association between performance and higher number of days antimicrobials were used (see Figure 5).

## 3. Discussion

### 3.1. Policy and Social Drivers of Antimicrobial Use

Indonesia has observed a move towards the production of broilers through integrated production companies and away from more independent smallholder production systems; a trend which is likely to continue [34]. These contract companies act as decision makers for antimicrobials routinely administered on the farm such that contract farmers have less control over antimicrobial use when compared with independent producers. 

Poor awareness of AMR has been identified as a hurdle to changing antimicrobial practices in many low- and middle-income countries [38,39]. Overall, study respondents had a poor knowledge of what defined AMR and expressed few concerns over the impact of AMR on either human or animal health. Within the respondent group, contract farmers reported fewer concerns over AMR when compared with independent producers. This finding highlights the lack of control that contract farmers have over antimicrobial use decisions and may reflect an absence of effective communication between contract companies and their contract producers with regard to antimicrobial use. Om et al. [40] observed, however, that there was no guarantee that antimicrobials were more effectively utilised on a contract farm in comparison to an independent holding. There is a need to encourage active dialogue between contract companies and farmers on promoting prudent antimicrobial use.

In many areas access to veterinarians is limited [27], consequently many farmers seek advice on antimicrobials from para-veterinarians, drug/medicine sellers and other farmers. Despite the poor availability of veterinary advice, the majority of respondents value the role of veterinarians as actors in monitoring the responsible use of antimicrobials. In human medicine it has been shown that regions with a lower number of physicians per head of population are more likely to observe higher non-prescription and inappropriate antimicrobial use [41,42]. Therefore, the limited availability of qualified veterinarians may be a barrier to the promotion of responsible antimicrobial stewardship programs in Indonesia. There is a need to increase access to veterinarians if the government is to limit veterinary antimicrobial use to prescription only.

Farmers currently rely heavily on para-veterinarians for advice on antimicrobial use, however, at present, the para-veterinarian training is predominantly vocational and does not include any specific training on responsible antimicrobial use. Yusuf et al. [43] identified that much of the antimicrobial use by government para-veterinarians in cattle may be inappropriate and that antimicrobials are commonly used for viral or parasitic conditions. This highlights a need for additional training for para-veterinarians on appropriate antimicrobial use. 

In response to global pressure Indonesia introduced legislation to prohibit the use of antimicrobial growth promoters (AGPs) in animal feed in January 2018 [27,44]. This policy presents a number of challenges both in terms of the enforcement but also in the interpretation of the legislation. There is no clear distinction between the practice of the administration of AGPs or the implementation of a routine antimicrobial prevention program [45,46]. Both uses have been associated with antimicrobial administration at low doses and for longer durations than for therapeutic indications; a practice which has been associated with the selection of resistant bacteria [9,18]. This blurred line between antimicrobial use for disease prevention or growth promotion, presents a challenge to the enforcement of the AGP ban. This barrier is more substantial in the current policy landscape with antimicrobials readily available over the counter without a veterinary prescription.

Economic incentives in terms of reduced feed, labour and capital are the primary drivers for the widespread use of AGPs, as the financial benefits often outweigh the costs of buying medicated feed [19]. Concerns have been expressed that the AGP ban will result in higher mortality rates as presently the use of antimicrobials in feed is believed to overcome the significant disease burdens seen in open broiler housing systems [47]. Farmers in the study valued the economic role of antimicrobials on their farm through their perceived role in reducing mortality and promoting growth rates. Thus, with the present structure of the broiler industry it is likely to require significant investment in order to mitigate for the economic losses from the ban on AGP. 

Further policies or regulations of antimicrobial use in livestock need to consider the current scale of domestic broiler production whereby, in parallel with the respondents’ farms, the majority of smaller commercial units are predominantly open housing systems [23]. These systems are vulnerable to the introduction of disease pathogens with biosecurity presenting a particular challenge and birds exposed to extreme temperatures and climatic factors. These systems have also been associated with higher mortality rates and health-related costs in comparison to closed housing systems [35]. With the process of intensification of broiler production in Indonesia there has been a move towards closed automated housing systems for larger-scale production systems [23]. These systems offer farmers superior options for preventing the introduction and spread of disease on farms. 

The WHO advocates the use of alternative methods to antimicrobial use such as improved animal housing and hygiene, more targeted use of vaccinations and the use of evidence-based husbandry practices [8]. In intensively-managed pigs, methods such as improving water quality, feed safety and selective breeding for disease resistance have been advocated as alternative routes to prevent disease [48,49]. The study results show the vulnerability of the Indonesian broiler sector to clinical disease with the majority of farms being open housing systems, which present major challenges in maintaining high standards of biosecurity or implementing effective hygiene practices to prevent the introduction and circulation of pathogens. In the face of withdrawing and reducing routine antimicrobial use there is a need to improve the robustness of broilers through improved management, husbandry and genetics. Therefore, at present the small and medium broiler sectors present a number of challenges with regards to disease prevention and control. There are concerns that any regulation to restrict access to antimicrobials may result in some farms not being economically viable.

At present, there are many questions over the capacity and economic feasibility of enforcing antimicrobial use policy in Indonesia; it is useful to draw on the experiences of neighbouring countries. For example, despite Thailand having a more extensive infrastructure and tighter control over antimicrobials, researchers have identified that the illegal use of antimicrobials in livestock may be widespread [39,50]. In order to minimise the risks of antimicrobials becoming a black-market commodity, a stepwise approach to regulation would be best taken in Indonesia. This would require increasing capacity to support the livestock sectors in a transition towards reduced antimicrobial use by the government, increased industry investment in modern closed housing systems and greater engagement with education initiatives on AMR. The end goal of this step-by-step process would be the restriction of antimicrobials for use in animals to a prescription-only status [44]. 

### 3.2. Economic Drivers of Antimicrobial Use

In the current economic climate there is strong competition between livestock production companies, with little incentive to reduce antimicrobial use, particularly as it may be associated with significant economic losses for the private sector [34,51,52]. The study results revealed that contract farmers more frequently identified an economic advantage to using antimicrobials when compared with independent farmers. Thus, the potentially high costs of reducing antimicrobial use may present a barrier to changing farmer behaviours. However, with increasing international pressure to address AMR there is a need to engage with both the private and public sectors; this is a strategic aim of the AMR action plan for Indonesia [24]. The process to achieve collaborative discussion between the public and private sectors on antimicrobial use in broilers has been initiated with meetings including both government representatives, broiler production companies, the pharmaceutical sector and other key stakeholders in poultry health on the subject of AMR [53]. 

The economic analysis has identified that medicines are a very small component of the total costs in broiler production ranging from 0.7% to 4.3% of total costs. This supports the results of studies on small commercial broiler systems in Vietnam [54]. The small relative cost of antimicrobials may be leading to an indiscriminate and overuse of antimicrobials in the broiler sector in Indonesia. The fact that they are easy to access and cheap means that farmers do not need to make antimicrobial use decisions based on price, but rather on their perceptions of the potential benefits. However, the study also identified contrasts in the cost structures in different provinces. For example, the initial cost analysis identified that overall farmers in West Kalimantan, particularly the small-scale independent farmers, have higher cost structures when compared with those in other areas. 

There may be many reasons for these provincial cost differences, such as distance from input markets, the size of the farm and management systems. Close geographic proximity to other broiler farms and a large urban market, have been associated with lower farm management costs for broiler producers [55]. West Kalimantan is a significant distance from the main input producing and marketing areas which are centred around Jakarta. This distance from the source of feed and DOCs would inevitably lead to higher transport costs. While there is only a relatively small sample in Lampung Province, the close proximity to West Java (including Jakarta) is likely to ensure that the main costs of DOCs and feed are relatively low in comparison in this area. Another potential reason for differences in cost between provinces may be contrasts in farm size. For example, the farms in West Kalimantan were generally smaller (median flock size 2000) in comparison to either Lampung (8000) or Central Java (5000) provinces. Smaller farms may not have the economies of size when it comes to DOCs and feed purchase. Additionally, contract companies and poultry shops are less likely to give discounts to smaller farms [56]. 

The second part of the economic analysis compared productivity, management systems and farm sizes to antimicrobial usage (estimated as days antimicrobials are used). The analysis indicated that there may be some productivity benefits to using antimicrobials. For example, while there may be a negative relationship between antimicrobial cost and productivity, there may be a positive relationship between antimicrobial use and productivity. The results showed that there may be a higher production with more days of antimicrobial use. 

## 4. Materials and Methods 

### 4.1. Study Design and Setting

The study was a collaboration between the University of Liverpool, the Food and Agriculture Organization of the United Nations (FAO) Country Office for Indonesia and the Center for Indonesian Veterinary Analytical Studies (CIVAS). The study population was small and medium commercial broiler producers who were established for at least two years. A convenience sample was taken guided by the local government veterinary service officers (VSOs) in the Indonesian provinces of Lampung (one district), Central Java (three districts) and West Kalimantan (ten districts). Farm selection was based on the size of the flock in relation to other broiler farms in the province and the accessibility of the farm for undertaking data collection. There was some variation in farm size between provinces, for example, West Kalimantan had smaller-sized farms in comparison to Lampung. The geographical location of the sample farms included in the two surveys by provincial and district levels is shown in Table 4.

### 4.2. Questionnaire

The questionnaire was developed by a team of researchers from the University of Liverpool, FAO and CIVAS, including local government veterinarians, veterinary epidemiologists, agricultural economists and a researcher familiar with qualitative studies into antimicrobial use behaviours. Its content was based on a previous FAO-funded knowledge, attitudes and practices (KAP) study undertaken in broiler production in Indonesia [53] and questionnaire studies into antimicrobial use practices in pigs undertaken in Vietnam, Thailand and the UK [57,58]. The questionnaire was developed in English and was translated into Bahasa Indonesian by researchers at CIVAS.

The questionnaire consisted of the following sections:

1. Demographic farm information.
  Demographic information on the type of broiler production, the workers employed on the farm, the flock size, location of the farm, feeding practices, management practices and productivity data on the broiler production enterprise.
2. The behavioural influences behind antimicrobial use and farmers’ perspectives on AMR.
  Farmers’ perceptions on the definition of AMR, drivers for antimicrobial use in broiler production, attitudes on the responsibility of antimicrobial use practices and the costs and benefits of antimicrobial use.
3. Survey on the economic drivers for antimicrobial use.
  The economic questions explored the profitability of the broiler enterprise, antimicrobial costs, feed costs, prices obtained for selling birds, average body weight at slaughter, weight at point of sale, mortality rates and other medicine management costs.

### 4.3. Data Collection

The questionnaire was conducted as a face-to-face structured interview on a single farm visit between August and October 2018 by provincial government VSOs familiar with broiler production systems. Training for VSOs was provided by the FAO to ensure uniformity in data collection techniques. Data were entered in Bahasa Indonesia and translated into English by researchers at CIVAS.

### 4.4. Data Analysis

Data analysis was undertaken using Microsoft Excel 2016 (Microsoft Corporation, Redmond, Washington, USA) and SPSS Statistics 24 (IBM SPSS Statistics for Windows Version 22.0. Armonk, NY, USA: IBM Corp). Descriptive statistics were presented as percentages of response categories or Likert scale responses. A Chi-squared test was used to determine significant differences in responses between independent farmers and those producing broilers under contract for production companies (the contrasts between contract and independent farmers are described in further detail in Section 2); *p*-values <0.05 were deemed significant. 

The economic analysis included two parts: The first was an analysis of production costs which compared cost structures between provinces, district, farm size and management system (contract or independent). The second discussed the relationships between productivity and location, farm size and antimicrobial costs and use. The economic analysis was undertaken in Microsoft Excel 2016 (Microsoft Corporation, Redmond, Washington, USA). This analysis evaluated the correlation between variables by estimating the coefficient of determination (R^2^). A high R^2^ signifies a strong relationship between variables and low R^2^ indicates a weak relationship between the variables. In an analysis such as this, with estimated data rather than measured data, a high R^2^ (e.g., above 90%) would not be expected.

### 4.5. Ethical Approval

Overall ethical approval was granted by the University of Liverpool Veterinary Science Research Ethics Committee which also required proof of local (country-level) ethical acceptability (reference 635 number VREC640). As the study did not involve the collection of samples from animals or humans, the research collaborators and local government in Indonesia did not require a specific ethical review. Therefore, documentation mitigating the need for a detailed ethical review was provided from the Directorate General of Livestock and Animal Health Services (DGLAHS) in Indonesia. 

## 5. Conclusions

Internationally there are examples where significant reductions in antimicrobials have been possible without negative effects on livestock productivity. For example, since the 1990s, Denmark achieved a reduction in antimicrobial use of around 60% in parallel with a 50% increase in productivity in the region. However, this success was centred on three key components: the collection of accurate and comprehensive data on antimicrobial use; political enforcement of regulations; and a collaborative approach between farmers, veterinarians, government and researchers [59]. These features are all currently absent from the Indonesian broiler sector.

With growing international pressure to reduce antimicrobial use in livestock, Indonesia faces significant challenges in all sectors; therefore it is essential that efforts are made to seek alternative methods of controlling disease to the routine administration of antimicrobials [27]. These challenges are outlined in Table 5. The Indonesian broiler sector requires detailed information on the economic viability of interventions to reduce antimicrobial use. This is particularly important in the small-scale broiler sector where margins are typically tight and farms are vulnerable to fluctuations in the market [60]. 

## Figures and Tables

**Table 1:** Demographic information on the respondents and the respondent farm.

		**Province Locations of Respondent Farms**							
		**Lampung Province**		**Central Java Province**		**West Kalimantan Province**		**All Provinces**	
**Respondent Characteristics**									
		***n***	**%**	***n***	**%**	***n***	**%**	***n***	**%**
**Role on farm**	**Farm owner**	29	63	126	80	250	85	405	82
	**Farm manager**	17	37	31	20	43	15	91	18
**Gender**	**Male**	48	94	152	94	277	95	478	94
	**Female**	3	6	10	6	15	5	28	6
**Education level**	**Junior school**	10	20.4	17	10.4	82	28.7	109	21.9
	**High school**	28	57.2	124	76.1	185	64.7	337	67.7
	**University**	11	22.4	22	13.5	19	6.6	53	10.4
		**Median**	**IQ range**	**Median**	**IQ range**	**Median**	**IQ range**	**Median**	**IQ range**
**Age**		42	15	42	12.5	41	14	42	14
**Years of experience working with broiler chickens**		8	8	5	6	5	5	5	6

IQ range: interquartile range.

**Table 2:** Demographic information on the respondent farms.

		**Province Locations of Respondent Farms**							
		**Lampung Province**		**Central Java Province**		**West Kalimantan Province**		**All Provinces**	
		***n***	**%**	***n***	**%**	***n***	**%**	***n***	**%**
**Farm type**	**Contract**	40	78	159	97	82	28	281	56
	**Independent**	11	22	5	3	206	72	222	44
**Housing type**	**Open sheds**	48	96	153	94	286	99	487	97
	**Closed sheds**	2	4	9	6	2	1	13	3
		**Median**	**IQ range**	**Median**	**IQ range**	**Median**	**IQ range**	**Median**	**IQ range**
**Current broiler population**		7450	9125	5000	2500	2000	2700	3000	4000
**Farm capacity**		8000	10,000	5000	2625	3000	4000	4250	4500
**Number of broiler production cycles per year**		6	1	6	0	6	1	6	1

IQ range: interquartile range.

**Table 3:** Average broiler production costs (United States Dollar (USD)/1000 birds) at a province and district level. The totals for each province (Central Java, West Kalimantan and Lampung) are shown in bold whilst the districts level data are presented below each province.

Province (Bold) and District of Respondent Farms	No.	DOC *	Feed	Disinfectant	Litter	Medicines	Labour	Heating	Other	Vaccine	Total
**West Kalimantan**	**293**	**569**	**1586**	**7**	**10**	**19**	**61**	**12**	**42**	**13**	**2320**
Ketapang	15	580	2262	31	11	87					2971
Mempawah	60	587	2131	5	8	12	78	10		7	2837
Kota Pontianak	40	595	1557	4	9	20	58	10	30	29	2312
Kubu Raya	60	567	1398	14	18	29	55	14	89	18	2202
Kayong Utara	15	667	1472	6		17			8	3	2173
Sambas	15	501	1481	2	13	18	53	8		12	2087
Sanggau	15	493	1401	3	10	14	65	25		0	2012
Kota Singkawang	60	535	1228	4	6	14	49	11		5	1853
Sekadau	13	596	1033	8	7	48	53	14		28	1788
**Central Java**	**165**	**474**	**1435**	**5**	**18**	**31**	**33**	**20**	**18**	**12**	**2046**
Semarang	45	456	1620	4	22	30	34	19	15	13	2213
Boyolali	60	469	1633	2	12	29	30	24		5	2205
Klaten	60	490	1287	6	17	35	32	21	21	21	1929
**Lampung**	**51**	**469**	**1246**	**1**	**9**	**30**	**34**	**17**	**17**	**42**	**1865**
Lampung Selatan	51	469	1246	1	9	30	34	17	17	42	1865
**Average**		**536**	**1505**	**7**	**11**	**29**	**49**	**16**	**30**	**17**	**2188**

* DOC: day old chick.

**Table 4:** Geographical distribution of broiler farms included in the study.

Province	District	Survey on the Economic Drivers for Antimicrobial Use
Central Java	Boyolali	60
	Klaten	60
	Semarang	45
Lampung	Lampung Selatan	51
West Kalimantan	Kayong Utara	15
	Ketapang	15
	Kota Pontianak	40
	Kota Singkawang	60
	Kubu Raya	60
	Mempawah	60
	Sambas	15
	Sanggau	15
	Sekadau	13

**Table 5:** Key findings and policy recommendations from a study exploring the economic and behavioural drivers of antimicrobial use in broilers in Indonesia.

Evidence for the Economic and Behavioural Drivers of Antimicrobial Use	Policy Recommendations
The ease of access to antimicrobials for Indonesian broiler producers.	The study results identified a need for a stepwise approach to restrict antimicrobial use, which would be best achieved with better regulation of drug sellers and pharmacies as the initial stage with the ultimate goal of making antimicrobials a prescription-only drug. There is a risk that implementing major changes to policy may encourage farmers to source antimicrobials from black-market sources [39].
Poor access to trained veterinarians and para-veterinarians for small commercial broiler producers.	There is a need to increase the numbers of veterinary professionals in Indonesia as well as implement a more formal and structured training for para-veterinarians, with a particular focus on responsible antimicrobial use.
Dominance of the integrated poultry production companies.	It is essential that any efforts to promote antimicrobial stewardship are led using a top-down approach by the industry. Significant progress has been made in Thailand through an industry-led initiative to collect veterinary antimicrobial sales data [61].
Overall antimicrobial use is a relatively minor cost for broiler producers. At present many farms rely on antimicrobials to control endemic disease.	Whilst increasing the cost of antimicrobials may act as a deterrent to their use, it is essential that any policy considers the likely negative effects in terms of food supply and the livelihoods of these small-scale commercial producers.
Economic benefits in the form of improved productivity rates from their use were observed despite efforts by the Indonesian government to reduce antimicrobial use in livestock in-line with international efforts to safeguard human and animal health.	There is a need for further research on the cost-effectiveness of alternative methods of preventing disease and ensuring that feasible alternatives are easily available. Farmers must be incentivised to seek alternative approaches to prevent disease, such as vaccinations and improvements in management systems, including on-farm biosecurity.
Record keeping on farm productivity was generally poor or absent.	The importance of collecting accurate farm productivity data and undertaking economic assessments in any interventions to reduce antimicrobial use.
Open housing systems dominate small commercial broiler production and leave birds vulnerable to disease introduction and exposed to extreme temperatures.	Closed housing, offering producers better facilities to prevent and manage disease, provides scope to encourage producers to reinvest in their housing systems. This could only be achieved through the engagement of the broiler production companies and there is a need to offer incentives for contract farmers.
There is a need to improve the robustness of broilers through improved management, husbandry and genetics.	Improvements in water quality, feed safety and genetics are essential to reducing the reliance on antimicrobials for disease prevention.
Knowledge on AMR and its potential wider consequences was limited.	There is a need for greater knowledge exchange with farmers on the definition of AMR and the potential negative effects on human and animal health.

## Supplementary Materials

The following are available online at https://www.mdpi.com/2079-6382/9/4/154/s1, Table S1: Univariate Chi-squared analysis comparing the independent and contract farmers’ opinions on AMR and antimicrobial use practices in broilers in Indonesia. Table S2: Differentiating smaller-scale farms from larger-scale farms with regard to on-farm costs ($US/1000 birds). Table S3: Differentiating contract and independent farmers with regard to on-farm costs ($US/1000 birds).
