# Supplementary material for: The Costs, Benefits and Human Behaviours for Antimicrobial Use in Small Commercial Broiler Chicken Systems in Indonesia
Source: Antibiotics (Basel). 2020 Apr 1;9(4):154. doi: 10.3390/antibiotics9040154 (PMC7235826; doi:10.3390/antibiotics9040154)
Supplement: Supplementary file 1 [file antibiotics-09-00154-s001.pdf]

Supplementary Material

Table S1. Univariate Chi-squared analysis comparing the independent and contract farmers' opinions on AMR and antimicrobial use practices in broilers in Indonesia.

		Independent farmer	Contract farmer	Chi-squared *p*-value
	**Ability to correctly define AMR**			
**Knowledge on AMR definition**	Unable to correctly define AMR	117 (52.7%)	173 (61.6%)	**0.046**
	Able to correctly define AMR	105 (47.3%)	108 (38.4%)	
	**The importance of AMR concerns and the role of antimicrobial use in broilers**			
**AMR will never be a problem on my farm**	Disagree	70 (31.7%)	100 (35.8%)	0.605
	Neutral	82 (37.1%)	95 (34.1%)	
	Agreed	69 (31.2%)	84 (30.1%)	
**AMR is a major human health concern in Indonesia**	Disagree	29 (13.1%)	38 (13.6%)	0.987
	Neutral	75 (33.9%)	94 (33.7%)	
	Agreed	117 (52.9%)	147 (52.7%)	
**Chickens are healthier if more antimicrobials are used**	Disagree	120 (54.3%)	101 (36.1%)	**<0.001**
	Neutral	46 (20.8%)	91 (32.7%)	
	Agreed	55 (24.9%)	87 (31.1%)	
**Antimicrobials used in chickens can affect the health of the consumer**	Disagree	23 (10.4%)	29 (10.4%)	0.648
	Neutral	73 (33.0%)	103 (36.9%)	
	Agreed	125 (56.6%)	147 (52.7%)	
**Antimicrobial use in animals should be reduced**	Disagree	15 (6.8%)	41 (14.7%)	**<0.001**
	Neutral	39 (17.6%)	104 (37.3%)	
	Agreed	167 (75.6%)	134 (48.0%)	
**Antimicrobial use in humans should be reduced**	Disagree	9 (4.1%)	26 (9.4%)	**<0.001**
	Neutral	35 (15.9%)	93 (33.5%)	
	Agreed	176 (80.0%)	159 (57.2%)	
**Indiscriminate use of antimicrobials can lead to AMR**	Disagree	12 (5.4%)	26 (9.4%)	0.26
	Neutral	75 (33.9%)	91 (32.7%)	
	Agreed	134 (60.6%)	161 (57.9%)	
**Many antimicrobials are not effective at treating disease in chickens**	Disagree	40 (18.1%)	60 (21.8%)	**0.014**
	Neutral	72 (32.6%)	115 (41.8%)	
	Agreed	109 (49.3%)	100 (36.4%)	
**Indiscriminate use of antimicrobials in chickens is expensive for farmers**	Disagree	6 (2.7%)	14 (5.1%)	**<0.001**
	Neutral	24 (10.9%)	73 (25.6%)	
	Agreed	190 (86.4%)	188 (68.4%)	
**There is a need for sufficient withdrawal time after antimicrobial use before selling birds for slaughter**	Disagree	5 (2.3%)	6 (2.2%)	**0.001**
	Neutral	26 (11.8%)	68 (25.1%)	
	Agreed	189 (85.9%)	199 (74.4%)	
	**Drivers for antimicrobial use in broiler flock**			
**To prevent disease in the flock**	Less likely	51 (23.4%)	45 (16.2%)	**0.036**
	Neutral	94 (43.1%)	112 (40.3%)	
	More likely	73 (33.5%)	121 (43.5%)	

**An increased mortality rate**	Less likely	32 (14.7%)	37 (13.3%)	0.872
	Neutral	70 (32.1%)	94 (33.8%)	
	More likely	116 (53.2%)	147 (52.9%)	
**The chickens are not eating**	Less likely	35 (16.1%)	42 (15.1%)	0.944
	Neutral	106 (48.6%)	139 (49.8%)	
	More likely	77 (35.3%)	98 (35.1%)	
**To improve productivity and growth in the flock**	Less likely	43 (19.7%)	36 (12.9%)	0.064
	Neutral	125 (57.3%)	161 (57.7%)	
	More likely	50 (22.9%)	82 (29.4%)	
**Advised by drug seller to use antimicrobials**	Less likely	23 (10.6%)	39 (14.1%)	0.075
	Neutral	131 (60.1%)	180 (65.0%)	
	More likely	64 (29.4%)	58 (20.9%)	
**Advised by the veterinarian or para-veterinarian to use antimicrobials**	Less likely	22 (10.1%)	21 (7.6%)	0.603
	Neutral	111 (50.9%)	144 (51.8%)	
	More likely	85 (39.0%)	113 (40.6%)	
**Advised by the production company to use antimicrobials**	Less likely	26 (11.9%)	25 (9.0%)	**<0.001**
	Neutral	148 (67.9%)	115 (41.5%)	
	More likely	44 (20.2%)	137 (49.5%)	
**Advised by another farmer to use antimicrobials**	Less likely	38 (17.5%)	46 (16.7%)	0.512
	Neutral	134 (61.8%)	183 (66.3%)	
	More likely	45 (20.7%)	47 (17.0%)	
**Role of key actors in monitoring the responsible use of antimicrobials in broilers**				
**Veterinarian or para-veterinarian**	Not important	7 (3.2%)	0 (0.0%)	**0.001**
	Neutral	21 (9.5%)	48 (17.2%)	
	Important	193 (87.3%)	231 (82.8%)	
**Farmer**	Not important	6 (2.7%)	5 (1.8%)	0.367
	Neutral	36 (16.3%)	58 (20.8%)	
	Important	179 (81.0%)	216 (77.4%)	
**Government**	Not important	4 (1.8%)	12 (4.3%)	**<0.001**
	Neutral	19 (8.6%)	57 (20.3%)	
	Important	198 (89.6%)	210 (75.3%)	
**Retailer**	Not important	22 (10.0%)	45 (16.4%)	**0.009**
	Neutral	62 (28.2%)	96 (35.0%)	
	Important	136 (61.8%)	133 (48.5%)	
**Economic importance of antimicrobial use for broiler farmers**				
**Does a health issue in the broiler flock have a negative effect on the farm profit margin?**	No	26 (11.8%)	32 (11.6%)	0.927
	Yes	194 (88.2%)	245 (88.4%)	
**Does the farm collect any productivity data on the broiler flock?**	No	38 (39.2%)	64 (43.0%)	0.557
	Yes	59 (60.8%)	85 (57.0%)	
**Is there an economic advantage to antimicrobial use?**	No	63 (28.6%)	24 (8.7%)	**<0.001**
	Yes	157 (71.4%)	252 (91.3%)	

Table S2. Differentiating smaller-scale farms from larger-scale farms with regard to on-farm costs ($US/1000 birds).

	No.	DOC	Feed	Disinfectant	Litter	Medicines	Labour	Heating	Other	Vaccine	Total
**Smaller farms**	255	570	1638	11	12	22	66	13	40	12	**2384**
Central Java	33	474	1401	41	16	35	37	20	15	5	**2043**
Lampung	8	508	952	2	13	21	69	21	0	64	**1651**
West Kalimantan	214	583	1665	7	12	21	71	13	58	12	**2442**
**Larger farms**	254	492	1381	10	13	23	35	16	19	16	**2005**
Central Java	132	474	1442	17	18	30	32	20	18	15	**2067**
Lampung	43	463	1266	1	9	31	31	17	17	20	**1855**
West Kalimantan	79	525	1349	5	7	12	43	12	26	17	**1996**
**Average**		**536**	**1522**	**11**	**12**	**22**	**47**	**15**	**22**	**14**	**2201**

Table S3. Differentiating contract and independent farmers with regard to on-farm costs ($US/1000 birds).

	No.	DOC	Feed	Disinfectant	Litter	Medicines	Labour	Heating	Other	Vaccine	TOTAL
**Independent**	**222**	**568**	**1668**	**6**	**10**	**19**	**61**	**11**	**13**	**13**	**2370**
Central Java	5										
Lampung	11	513	1071	2	12	21	59	18	7	42	1745
West Kalimantan	206	572	1680	7	10	18	62	11	15	13	2387
**Contract**	**281**	**503**	**1377**	**2**	**14**	**28**	**36**	**18**	**23**	**15**	**2017**
Central Java	159	476	1436	3	18	32	33	20	18	13	2049
Lampung	40	460	1272	1	9	31	31	17	27	0	1848
West Kalimantan	82	557	1333	8	11	22	56	15	70	18	2089

Note: six farmers, one in Central Java and five in West Kalimantan did not identify as either contract or independent farmers. The sample of five independent farmers in Central Java was insufficient to elicit representative data.
